# Quercetin Induces Macrophage Polarization through PI3K-AKT Pathway in Atherosclerosis: Network Pharmacology, Molecule Docking and in vitro Analysis

**DOI:** 10.5812/ijpr-168202

**Published:** 2026-02-18

**Authors:** Li Wang, Yifan Han, Yuyu Lei, Jingfeng Liu, Jiajun Yu, Guiyu Li

**Affiliations:** 1 The Eighth Affiliated Hospital, Sun Yat-Sen University, Shenzhen, China; 2 Qinghai University, Xining, China; 3 Peking University Shenzhen Hospital, Shenzhen, China

**Keywords:** Atherosclerosis, Macrophage Polarization, Traditional Chinese Medicine, Anti-inflammation, Quercetin

## Abstract

**Background:**

Quercetin (QU) is a major flavonoid in multiple herbs with wide biological effects, while its role in preventing atherosclerosis progression remains largely unknown.

**Objectives:**

In this study, we aimed to uncover the effect and underlying mechanism of QU in treating atherosclerosis.

**Methods:**

Network pharmacology and protein-protein interaction analysis were conducted to predict potential targets of QU in treating atherosclerosis. Gene ontology (GO) enrichment and Kyoto Encyclopedia of Genes and Genomes (KEGG) pathway enrichment analysis were performed. Cell viability, apoptosis, and cell cycle assays were conducted to evaluate the biological effects of QU on atherosclerosis-associated macrophages. Genome expression sequencing (RNA-seq) was performed to identify differentially expressed genes and regulated pathways after QU treatments. Macrophage differentiation and fluorescence-activated cell sorting (FACS), western blot, and reverse transcription quantitative polymerase chain reaction (RT-qPCR) were further performed to verify the results of RNA-seq and the effects of QU on regulating the indicated pathway.

**Results:**

In this study, 180 potential targets were identified by network pharmacology analysis, and multiple anti-inflammatory pathways were enriched in QU treating atherosclerosis. Our data also indicated that QU promotes macrophage cell viability by reducing cell apoptosis and cell cycle arrest in an atherosclerosis-associated macrophage cell model. Additionally, RNA-seq revealed that the PI3K-AKT pathway might be the significantly upregulated pathway after QU treatment in macrophages to induce M2 polarization, which was further verified by protein and RNA detection.

**Conclusions:**

Taken together, we are the first to combine multiple database analyses and genome expression data to uncover the protective effect of QU in treating atherosclerosis by inducing M2 polarization through regulating the PI3K-AKT pathway.

## 1. Background

Atherosclerosis, a chronic inflammatory disease of the arterial wall, is a leading cause of cardiovascular diseases, which account for the majority of deaths worldwide (1-3). Epidemiological studies reveal that atherosclerosis contributes to nearly 50% of all deaths in developed countries and is becoming increasingly prevalent in developing nations, including China, due to aging populations and lifestyle changes (4). The pathogenesis of atherosclerosis is closely linked to dysregulated immune responses, involving both innate and adaptive immunity (5). Endothelial dysfunction, triggered by factors such as hyperlipidemia and oxidative stress, initiates the recruitment of monocytes into the arterial intima, where they differentiate into macrophages. These macrophages engulf oxidized low-density lipoprotein (Ox-LDL) to form foam cells, a hallmark of early atherosclerotic lesions (5, 6). The innate immune system is further activated through pattern recognition receptors (PRRs), such as Toll-like receptors (TLRs), which recognize damage-associated molecular patterns (DAMPs) and amplify inflammation. Adaptive immunity also plays a critical role, with T-cells infiltrating plaques and releasing pro-inflammatory cytokines, such as interferon-γ (IFN-γ), which exacerbate vascular inflammation and plaque instability. This interplay between innate and adaptive immunity drives the chronic inflammatory state that underpins atherosclerosis progression (7, 8).

Despite advances in lipid-lowering therapies, such as statins, and anti-inflammatory treatments, these approaches often fail to fully address the complex immunopathology of atherosclerosis. This has prompted growing interest in alternative therapeutic strategies that target immune regulation. Traditional Chinese Medicine (TCM) offers a rich repository of bioactive compounds with immunomodulatory properties that may complement existing treatments (9, 10). For instance, Salvia miltiorrhiza (Danshen) has been shown to suppress inflammatory signaling pathways, including TLR-mediated responses, thereby reducing the production of pro-inflammatory cytokines (11). Astragalus membranaceus (Huangqi) has demonstrated the ability to modulate immune cell activity, including the regulation of macrophage and T cell responses, while Panax notoginseng (Sanqi) exhibits anti-inflammatory effects by attenuating oxidative stress and immune activation (12, 13). These findings underscore the potential of TCM to provide multi-targeted approaches for managing atherosclerosis by addressing its immunological underpinnings.

Quercetin (QU), a natural flavonoid widely found in fruits, vegetables, and TCM formulations, for example onions (around 200-320 mg/kg) (14), has garnered attention for its diverse pharmacological properties, including anti-inflammatory, antioxidant, and immunomodulatory effects (15-18). Beyond its potential in atherosclerosis, quercetin has demonstrated therapeutic benefits in various diseases characterized by chronic inflammation and tissue remodeling. For instance, in interstitial fibrosis, QU has been shown to attenuate fibroblast activation and extracellular matrix deposition by modulating TGF-β/Smad signaling pathways (19). Similarly, in inflammatory bowel disease (IBD), quercetin exerts protective effects by suppressing pro-inflammatory cytokine production, such as TNF-α and IL-6, and by restoring intestinal barrier integrity (20). These findings highlight quercetin's ability to regulate key molecular pathways involved in inflammation and tissue repair. Mechanistic studies have further elucidated the molecular targets of quercetin, revealing its capacity to modulate signaling pathways such as NF-κB, cGAS-STING, Nrf2, etc., which are central to inflammatory and oxidative stress responses. Quercetin has also been reported to influence immune cell function, including B cell activation, T cell differentiation, and the inhibition of inflammasome activation (21-26). Despite these promising findings, the application of QU in atherosclerosis research remains relatively underexplored, particularly in the context of large-scale data-driven studies. To date, most investigations have focused on in vitro or small-scale in vivo models, leaving a significant gap in understanding its broader therapeutic potential in atherosclerosis. Our study aims to address this gap by leveraging multiple database analyzing approaches to systematically evaluate the role of QU in atherosclerosis, providing new insights into its clinical relevance and mechanisms of action.

Macrophage polarization plays a pivotal role in the progression and resolution of atherosclerosis. In particular, the shift toward an anti-inflammatory M2 macrophage phenotype has been associated with plaque stabilization and the prevention of atherosclerosis progression (8, 27). It has been suggested that QU may influence macrophage activation and reduce inflammatory responses within atherosclerotic plaques (28). However, whether the therapeutic effects of QU in atherosclerosis are directly mediated through macrophage polarization remains unclear. The underlying mechanisms of this interaction require further investigation to elucidate the role of QU in modulating macrophage function and its implications for atherosclerosis treatment.

## 2. Objective

In this study, we used network pharmacology analysis and protein-protein interaction analysis to predict the underlying targets of QU in the treatment of atherosclerosis. In addition, whole genome expression combined with in vitro experimental verifications further strengthens the results of data processing. In this case, our data suggest that QU exerts a protective role in the treatment of atherosclerosis by activating M2 macrophage polarization through regulating the PI3K-AKT pathway.

## 3. Methods

### 3.1. Sources of the Chemical

Quercetin (HPLC ≥ 98%, CAS: 117-39-5) was purchased from Push Bio-Technology (Chengdu, China).

### 3.2. Cell Culture

Raw264.7 macrophages were obtained from Procell Life Science & Technology (Wuhan, China, CAS: CL-0190) and tested for no mycoplasma contamination. Cells were maintained in high glucose DMEM (Gibco, USA) supplemented with 10% FBS (Gibco, USA) and 1% penicillin-streptomycin (Gibco) in a humidified incubator at 37°C and sub-cultured every 3 days with 1:3 dilution.

### 3.3. Cell Viability Assay

In brief, various working concentrations (0, 25, 50, 100, and 200 µM) of QU were added to the culturing medium with 100 µg/mL Ox-LDL (Yiyuan Biotechnology, Guangzhou, China) and incubated as indicated for 24 h. After incubation, the culture medium was discarded and replaced with normal medium supplemented with 10 μL CCK-8 reagent (Dojindo, CAS: CK04-01) followed by 1-hour treatment. Finally, treated plates were measured at 450 nm using a microplate reader. The ratio of cell viability was considered 100% in control cells.

### 3.4. Fluorescence-Activated Cell Sorting Detection

Raw264.7 macrophage cells were incubated with Ox-LDL with or without 100 µM QU for 24 h. After incubation, fluorescence-activated cell sorting (FACS) detection for indicated marker expression was initiated. For flow cytometry analysis, we incubated 1×10^6^ cells with primary antibodies at room temperature for 1 h. After washing with 1 mL DPBS (Gibco), stained cells were suspended with 400 μL FACS buffer and analyzed with a BD LSR Fortessa machine and BD FACSDiva Software version 8.0. Fluorescence-activated cell sorting antibodies used in this paper are listed as follows: PE anti-Nos2 (iNOS), FITC anti-mouse CD206 (MMR) Antibody (Biolegend, CAS: 696805, 141703).

### 3.5. Cell Cycle Analysis

Cell cycle analysis was conducted as mentioned before (29). Data processing was performed using MODFIT software.

### 3.6. Apoptosis Assay

To determine the biological effect of QU on macrophages, Raw264.7 cells were incubated with Ox-LDL with or without 100 µM QU for 24 h. After incubation, apoptosis assay was performed following the manufacturer's instructions (BD Pharmingen™ PE Annexin V Apoptosis Detection Kit, CAS:559763). Quantification was analyzed using BD FACSDiva Software (version 8.0) and FlowJo (version 10.8.1).

### 3.7. Reverse Transcription Quantitative Polymerase Chain Reaction

Total RNA was extracted after the indicated treatment and reversely transcribed into cDNA. Then gene expression level was determined by reverse transcription quantitative polymerase chain reaction (RT-qPCR). Primers used in this study are listed in Table 1. 

**Table 1.** The Primers Used in this Study.

Genes	Primers（5' - 3'）
**IL-6**	
F	CAAAGCCAGAGTCCTTCAGAG
R	GTCCTTAGCCACTCCTTCTG
**IL-17**	
F	TCCAGAATGTGAAGGTCAACC
R	TATCAGGGTCTTCATTGCGG
**TNF-α**	
F	CTTCTGTCTACTGAACTTCGGG
R	CAGGCTTGTCACTCGAATTTTG
**TGF-β**	
F	CCTGAGTGGCTGTCTTTTGA
R	CGTGGAGTTTGTTATCTTTGCTG
**IL-10**	
F	AGCCGGGAAGACAATAACTG
R	GGAGTCGGTTAGCAGTATGTTG
**β-actin (ms)**	
F	ACCTTCTACAATGAGCTGCG
R	CTGGATGGCTACGTACATGG

### 3.8. Western Blot Detection

Cells were seeded in 6-well culture plates (3.0 × 10^6^ cells/well) overnight, followed by treatment with indicated concentrations of Ox-LDL with or without QU for 24 h. After incubation, cellular protein was extracted and expression was detected using the following antibodies: Phospho-Akt (Ser473) Antibody #9271, Akt Antibody #9272, PI3K Antibody #AF6242, P-PI3K Antibody #AF3242, Bcl-2 (D17C4) Rabbit mAb #3498, Bax Antibody #2772, β-Actin Antibody #4967, GAPDH (14C10) Rabbit mAb #2118.

### 3.9. Network Pharmacology Analysis

The bioactive compounds of the herbal formulation (Quercetin, QU) were identified through a comprehensive literature review and database mining using resources such as the Traditional Chinese Medicine Systems Pharmacology Database (TCMSP), TCMIO, and ChEMBL database. Potential protein targets of QU were predicted using databases such as SwissTargetPrediction, STITCH, BindingDB, TCMIO, and ChEMBL database. Disease-related targets were retrieved from publicly available databases, including GWASDB, GWASCAT, GeneCards, OMIM, and DisGeNET. The compound-target and disease-target datasets were integrated to construct a compound-target-disease network using Cytoscape software. To explore the biological pathways involved, the hub targets were subjected to gene ontology (GO) and Kyoto Encyclopedia of Genes and Genomes (KEGG) pathway enrichment analysis using tools such as DAVID or the clusterProfiler R package.

### 3.10. RNA-Sequencing Analysis

For whole genome expression analysis, RNA-sequencing was performed and analyzed as described before (29). Briefly, total RNA from control, Ox-LDL, and Ox-LDL+QU treated groups was extracted and the sequencing libraries were established. Paired-end sequencing was performed (Illumina HiSeq system) to obtain the raw data. Clean reads were then generated and mapped to the Mus musculus genome (GRCm39) using hisat2 with default parameters. Differentially expressed genes (P < 0.05) were analyzed with DEseq2. KEGG and GO analysis were conducted by KOBAS3.0 (kobas.cbi.pku.edu.cn/kobas3), and pathways with P < 0.05 were considered to be statistically significant.

### 3.11. Molecular Docking Prediction

To elucidate the binding interactions between QU and its primary atherosclerotic targets, molecular docking simulations were conducted following a standardized computational workflow. The 3D structure of QU (CID: 5280343) was retrieved from the PubChem database and converted to PDB format using Open Babel (v2.3.2). High-resolution crystal structures of the target proteins, including AKT1 (PDB ID: 4GV1), JAK1 (PDB ID: 4E4N), HIF1-α (PDB ID: 3HQR), mTOR (PDB ID: 4JSV), and STAT3 (PDB ID: 6NJS) were obtained from the RCSB Protein Data Bank. Protein pre-processing was performed using PyMOL (v2.3.4) to remove water molecules and heteroatoms, followed by the addition of polar hydrogens and Gasteiger charges via AutoDockTools (v1.5.6). Docking was executed using AutoDock-Vina (v1.1.2). Binding affinities were evaluated based on the lowest docking score (kcal/mol), where a value less than -6.0 kcal/mol was considered indicative of high affinity. Finally, intermolecular interactions were visualized and analyzed using PyMOL and the Protein-Ligand Interaction Profiler (PLIP).

### 3.12. Statistical Analysis

Statistics were performed as we described previously (29). All the experiments were biologically repeated at least three times. All values presented are the mean ± SD. P < 0.05 and P < 0.01 are considered statistically significant.

## 4. Results

### 4.1. Network Pharmacology Analysis Discovers Potential Targets of Quercetin in Treating Atherosclerosis

**Figure 1.**
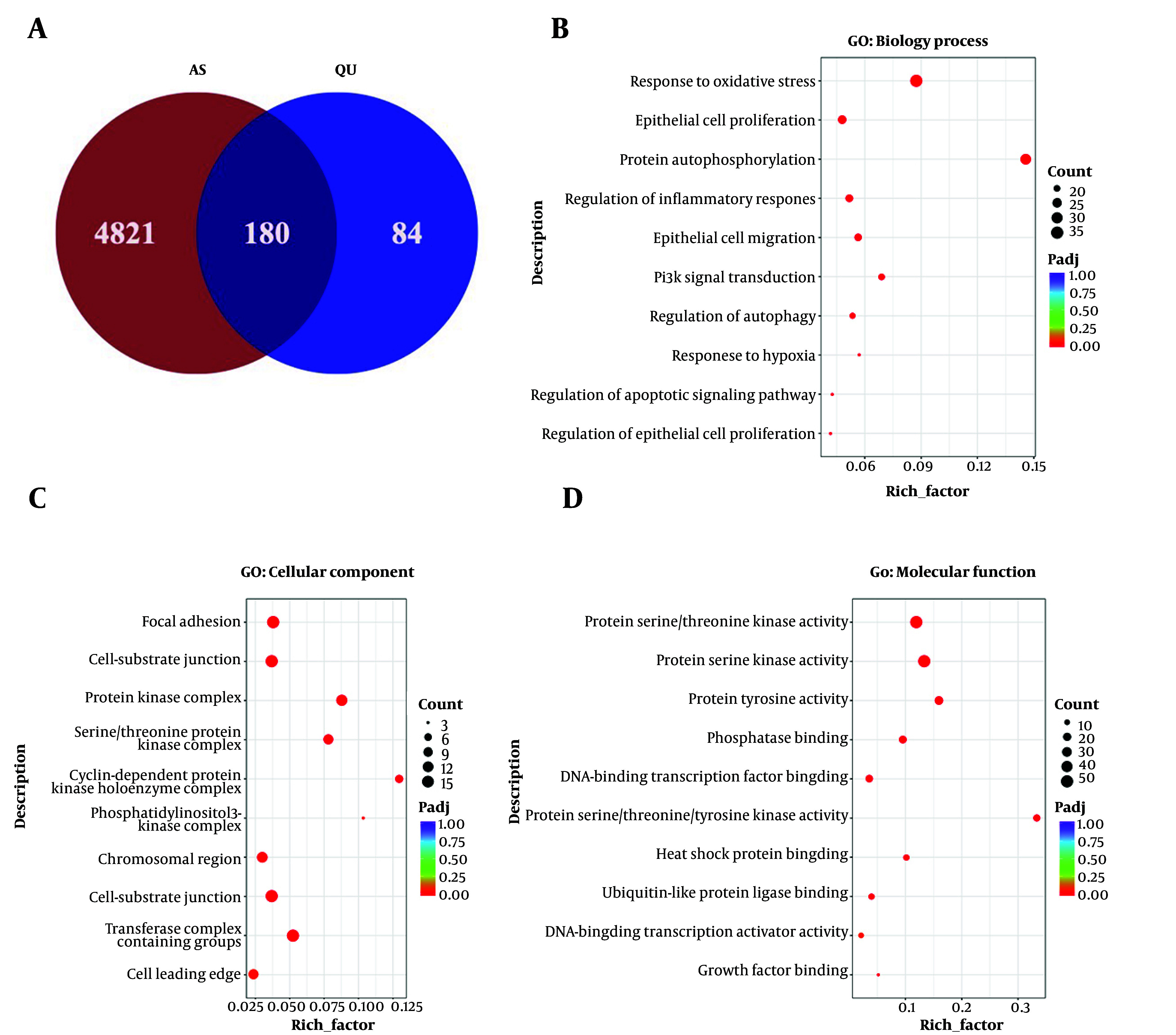
Network pharmacology analysis reveals potential targets of quercetin (QU) in the treatment of atherosclerosis (AS); A, the overlap in QU targets in CHEMBL and TCMIO database and differential genes of AS from BEFREE, LHGDN, CTD-human, HPO, RGD, TTD, GWASDB, WASCAT, and Genecards database; B-D, GO enrichment analysis of biological processes, cellular component, and molecular function with the overlapped targets; the X-axis of the histogram is the rich factor of individual terms and the Y-axis displays the GO categories.

Taking advantage of the emerging use of network pharmacology analysis tools, we can predict the potential targets of traditional Chinese herbs of interest for mechanism research. In this study, by searching relevant databases after importing the compound of interest, Quercetin (abbreviated as QU in this study), and applying the screening criteria of oral bioavailability (OB) ≥ 30% and drug-likeness (DL) ≥ 0.18, 264 targets were identified (Supplementary File 1). Furthermore, we identified 5001 targets by searching for the disease atherosclerosis (AS) across multiple databases (Supplementary File 2). The predicted 264 potential targets and 5001 AS-related gene sets were compared to identify the potential targets of QU for the treatment of AS. Finally, 180 target genes of QU for the treatment of AS were identified, as shown in Figure 1A. 

### 4.2. Gene Ontology Enrichment Analysis of Quercetin in Treating Atherosclerosis

Next, we carried out GO enrichment analysis on the 180 identified target genes. The GO enrichment analysis results, covering three categories — biological process, cellular component, and molecular function — revealed that AS-associated biological processes were significantly enriched. These included response to oxidative stress, epithelial cell proliferation, protein kinase complex, and protein kinase activity, which were among the top-ranked terms (Figure 1B-D). 

### 4.3. Potential Regulated Pathways of Quercetin in Treating Atherosclerosis

**Figure 2.**
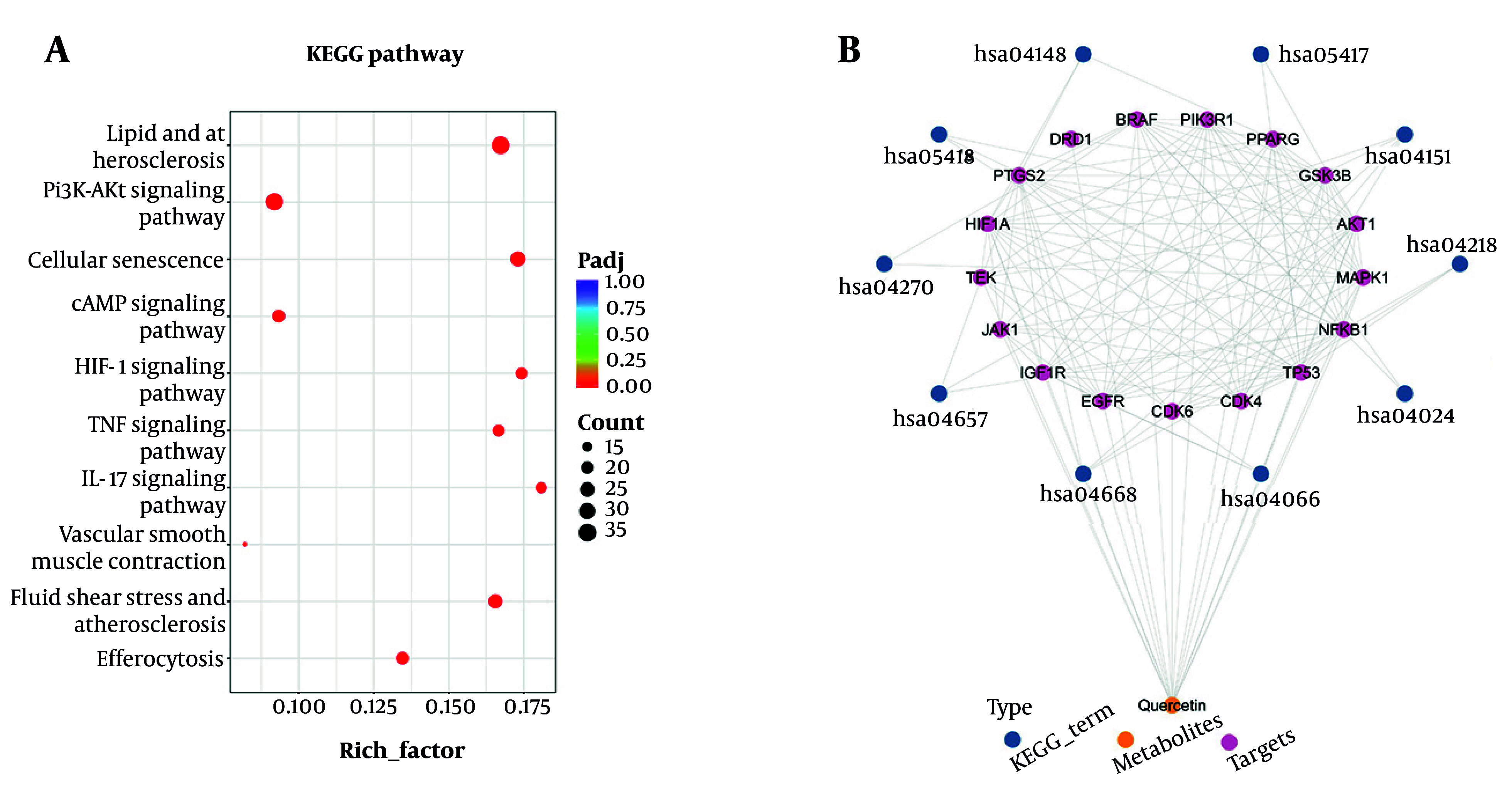
KEGG pathway enrichment and protein-protein interaction (PPI) analysis; A, the KEGG pathway enrichment analysis of overlapped targets; the X-axis of the histogram is the rich factor of individual terms and the Y-axis displays the top 10 KEGG categories among the results; B, the PPI network results of the top 17 important targets among the top 10 pathways with quercetin (QU) based on CytoNCA

Furthermore, the KEGG pathway enrichment analysis revealed that QU exerts its therapeutic effects on AS primarily through pathways such as lipid and atherosclerosis, PI3K-AKT signaling, cellular senescence, cAMP signaling, TNF signaling, and IL-17 signaling, as shown in Figure 2A. Additionally, by identifying the key proteins involved in these pathways, the protein-protein interaction (PPI) network analysis revealed close interactions between QU and key proteins, such as HIF1-α, JAK1, AKT1, and MAPK1, as shown in Figure 2B. Overall, the results demonstrated that the identified pathways and proteins were significantly affected by QU treatment during the progression of AS, suggesting that QU plays a critical role in the treatment of AS.

### 4.4. Molecular Docking Predicts Potential Binding Targets of Quercetin in Treating Atherosclerosis

**Figure 3.**
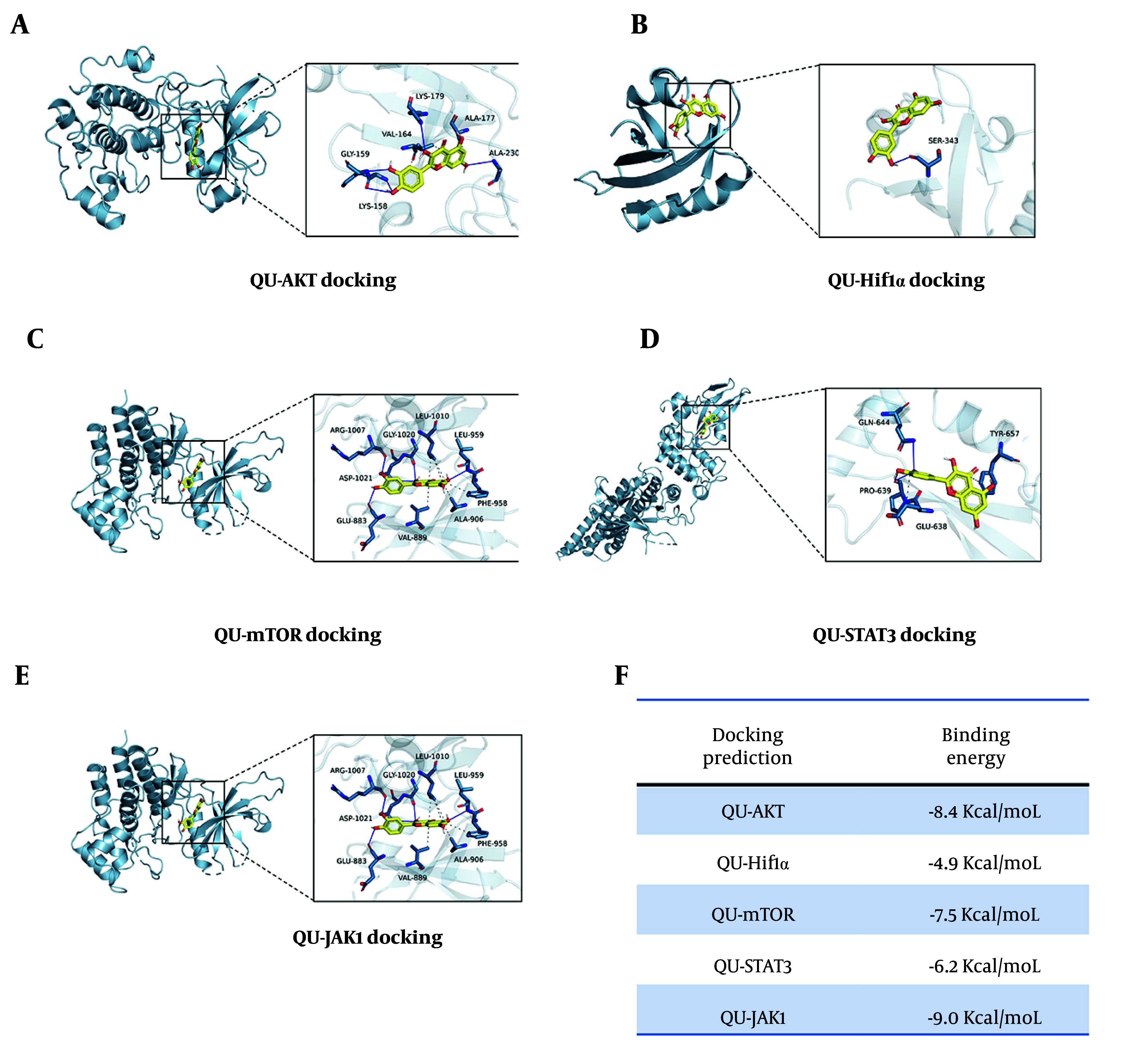
Molecular docking of underlying targets; A-E, molecular docking prediction of underlying targets of quercetin (QU) in atherosclerosis (AS) treatment; F, the predicted binding energy of indicated targets.

To predict the possible targets of QU in the treatment of AS, we identified key molecules based on the PPI results mentioned above and conducted molecular docking experiments. The required structure files were obtained from the PDB database. According to the results of molecular docking, a binding energy of ≤ -6 kcal/mol was considered indicative of good affinity with QU. As shown in Figure 3A-F, JAK1 and AKT proteins exhibited the strongest binding affinity to QU, a finding that aligns with the GO and KEGG analyses mentioned earlier. These results further indicate that QU can treat or improve the progression of AS but require further experimental validation.

### 4.5. Quercetin Plays Protective Role in Atherosclerosis Associated Macrophages

**Figure 4.**
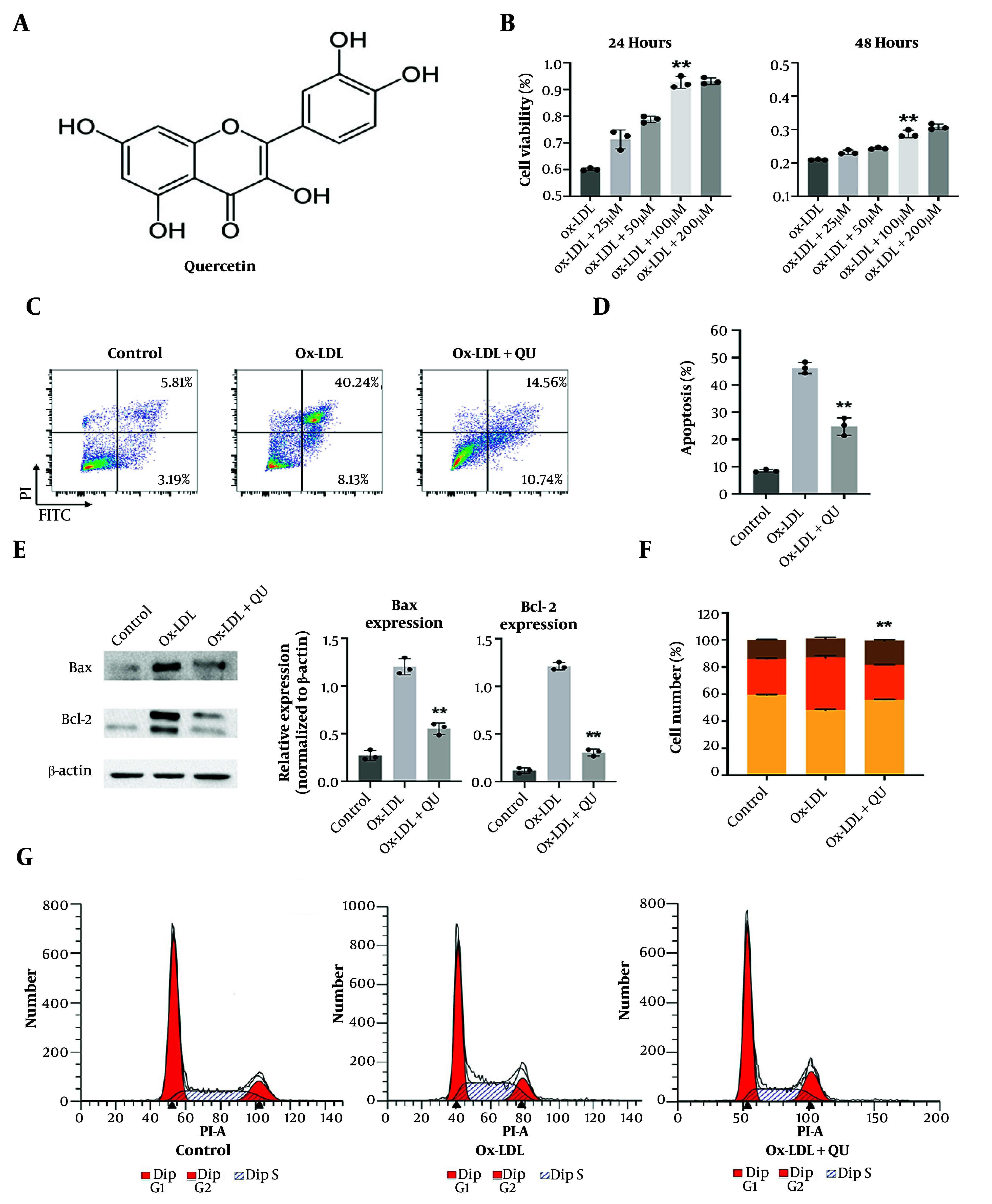
Biological functions of quercetin (QU) in atherosclerosis-related macrophages; A, the structure of Quercetin; B, cell viability analysis with different concentrations of QU in Ox-LDL treated Raw264.7 macrophages as the cell model of atherosclerosis (AS) progression; C, the apoptosis analysis of QU in Ox-LDL treated Raw264.7 macrophages; the representative fluorescence-activated cell sorting (FACS) images are displayed; D, statistical analysis of apoptosis assay, n = 3; E, the protein expression of Bax and Bcl-2 after QU treatment within Ox-LDL treated Raw264.7 macrophages; representative western blot results are shown and statistical analysis was calculated; F-G, cell cycle analysis of QU in Ox-LDL treated Raw264.7 macrophages; the representative cell cycle images are displayed; statistical analysis of cell cycle assay, n = 3; * P < 0.05, ** P < 0.01 vs Ox-LDL group

Macrophages are reported to play an important role in the activation and progression of AS. The above results from the network pharmacology analysis suggest that multiple pathways and key proteins are regulated or bound by QU. Notably, these pathways are closely related to macrophage functions. This raised the question of whether QU impacts macrophage biological processes. To address this, we conducted a cell viability assay using Ox-LDL-treated Raw264.7 macrophages, an established in vitro model of AS-associated macrophages. Quercetin supplementation increased the viability of Ox-LDL-treated macrophages, with the 100 μM concentration showing optimal effects after 24- and 48-hour incubation (Figure 4A-B). Furthermore, cell apoptosis, a form of programmed cell death, was also reduced following QU treatment (Figure 4C-D). Consistent with this, the protein expression levels of Bax and Bcl-2 further confirmed that QU protected macrophages from apoptosis (Figure 4E). In addition, since cell apoptosis can be induced alongside cell cycle arrest, cell cycle analysis was also performed under the same conditions. Consistent with the above results, QU treatment reduced cell cycle arrest at the S phase in stimulated macrophages (Figure 4F-G). These findings demonstrate for the first time that QU plays a critical role in treating AS by protecting macrophages from apoptosis and cell cycle arrest.

### 4.6. Quercetin Inhibits Multiple Proinflammatory Effects in Macrophages

**Figure 5.**
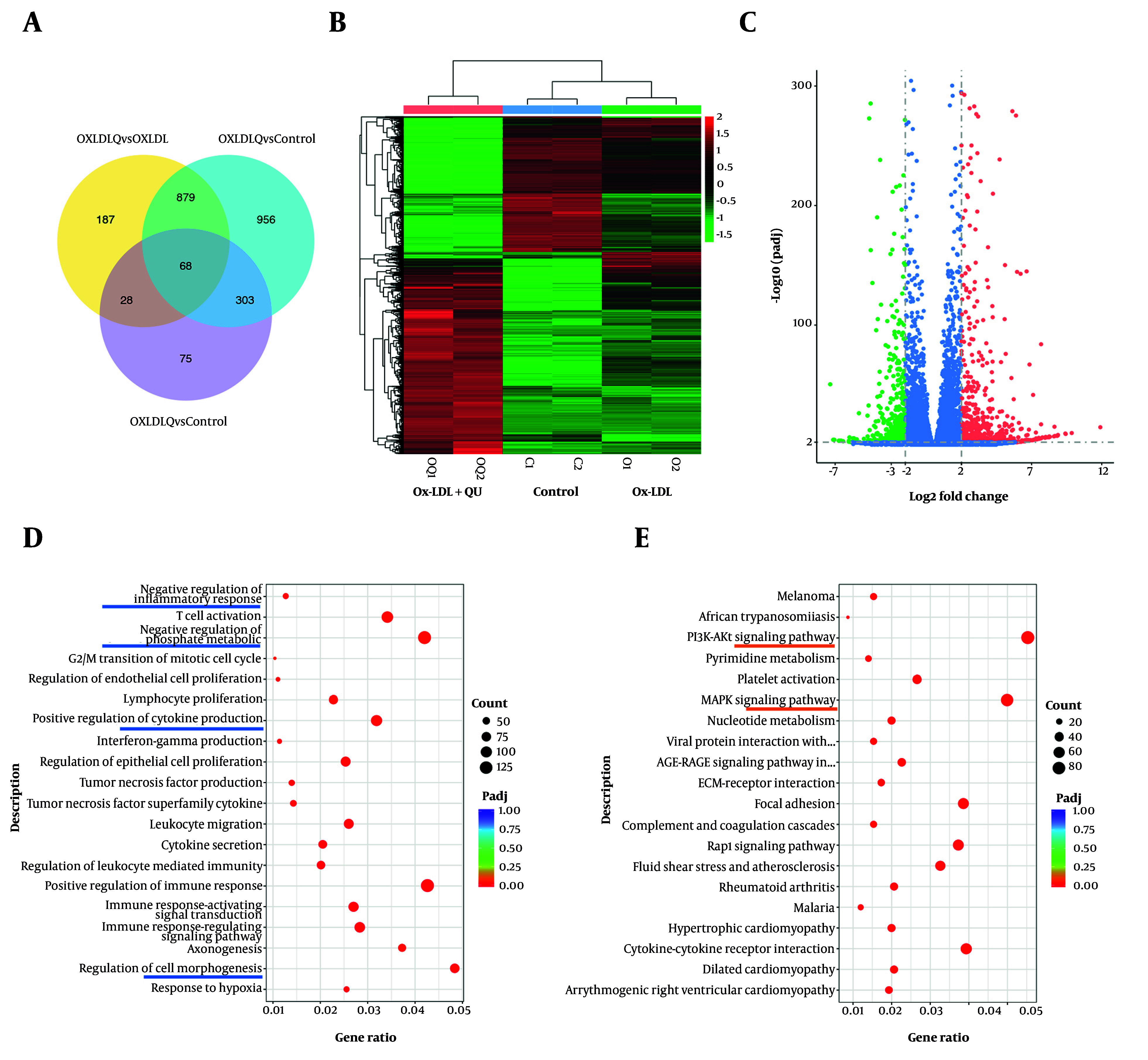
Quercetin (QU) regulates multiple pathways in macrophages; A, 68 genes were significantly regulated simultaneously in mutual comparison within control, Ox-LDL, and Ox-LDL+QU groups; B, the heatmap of differential gene expression among three groups; C, the volcano map of differential gene expression among the comparison between Ox-LDL and Ox-LDL+QU groups; D, GO enrichment analysis of differential genes among the comparison between Ox-LDL and Ox-LDL+QU groups; E, KEGG pathway analysis of differential genes among the comparison between Ox-LDL and Ox-LDL+QU groups

**Figure 6.**
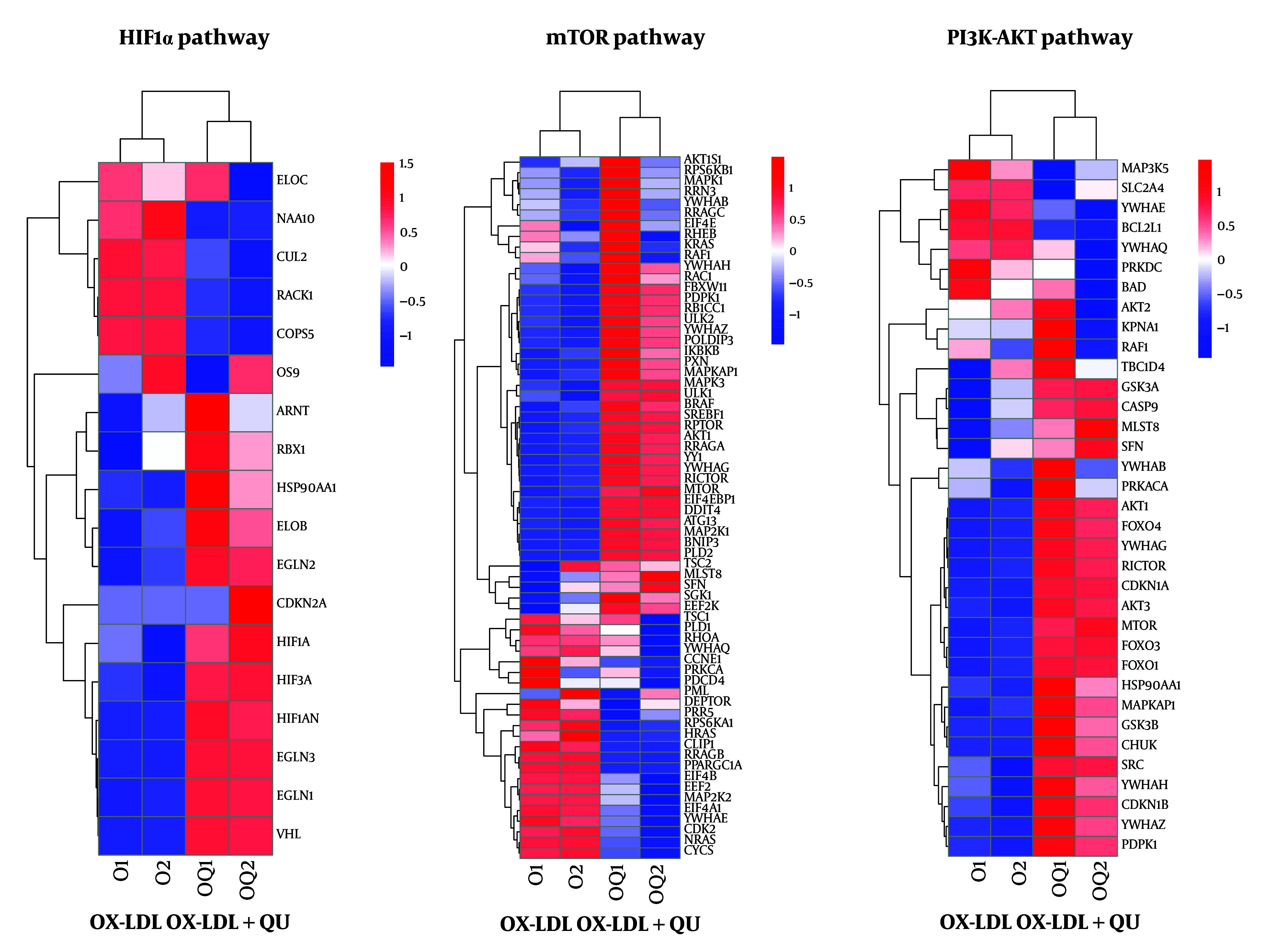
The transcriptional expression of significantly regulated genes involved in macrophage polarization. Significantly altered genes involved in Hif1α, mTOR, and PI3K-AKT pathways.

To further determine the underlying mechanisms regulated by QU treatment in AS-associated macrophages, we performed RNA sequencing to analyze global gene expression across three groups: Macrophages with no treatments, Ox-LDL-treated macrophages, and Ox-LDL-treated macrophages supplemented with QU. After processing the clean reads, 68 genes were identified as significantly modulated through comparative analysis, as shown in Figure 5A. The heatmap displayed differentially regulated genes among the three groups, and volcano plot analysis revealed that 1,931 genes were significantly upregulated, while 1,795 genes were significantly downregulated in the Ox-LDL-treated macrophages supplemented with QU (Figure 5B-C). In addition, GO enrichment analysis revealed that the regulated genes were primarily enriched in inflammation-related biological processes, such as the negative regulation of inflammatory response, negative regulation of phosphate metabolism, and positive regulation of cytokine production (Figure 5D). Furthermore, KEGG pathway analysis identified the PI3K-AKT pathway as one of the most enriched pathways, which aligns closely with the network pharmacology analysis data mentioned earlier (Figure 5E). To further investigate the pathways closely associated with inflammatory responses, HIF1α, mTOR, and the PI3K-AKT pathway were selected for differential analysis between Ox-LDL-treated macrophages and QU-supplemented Ox-LDL-treated macrophages (Figure 6). Our results demonstrated that genes in the PI3K-AKT pathway may be closely associated with the effects of QU treatment in AS.

### 4.7. Quercetin Induces M2 Polarization to Exert Protective Effect in Atherosclerosis via PI3K-AKT Pathway

**Figure 7.**
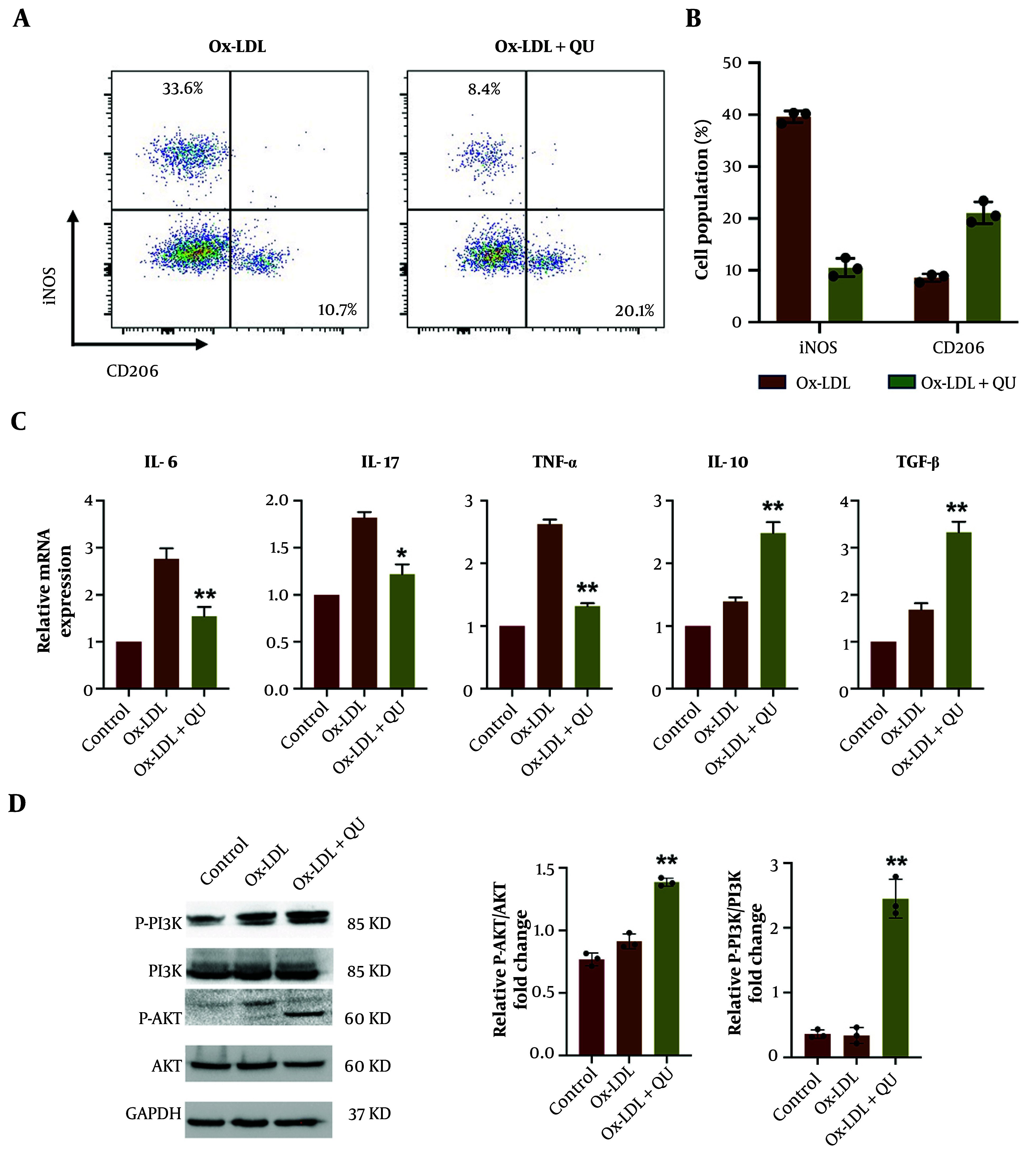
Quercetin (QU) promotes M2 macrophage polarization during AS progression via regulating PI3K-AKT pathway; A, macrophage polarization was determined by fluorescence-activated cell sorting (FACS) detection through labeling iNOS and CD206 indicating M1 and M2 macrophages respectively; B, statistical analysis for macrophage polarization FACS results; C, the mRNA detection of differentially expressed M1 and M2 specific genes; D, protein expression of AKT, p-AKT, PI3K, and p-PI3K in Ox-LDL and Ox-LDL+QU treated macrophages; representative western blot images are displayed; statistical analysis is displayed; n = 3; * P < 0.05, ** P < 0.01 vs Ox-LDL group

It is reported that macrophage polarization to the M1 phenotype promotes the progression of AS. Therefore, inducing M2 polarization is considered an effective strategy to treat AS. Accumulated data have identified that PI3K-AKT pathway activation plays a key role in triggering M2 macrophage differentiation. Consistent with this idea, FACS analysis to distinguish M1 and M2 macrophages, with or without QU treatment, showed that M2 polarization was induced, as evidenced by an increased proportion of CD206-positive macrophages (Figure 7A-B). In addition, RNA expression of IL-10 and TGF-β was upregulated, while IL-6, IL-17, and TNF-α were downregulated (Figure 7C), indicating the activation of M2 polarization following QU treatment. Furthermore, the protein expression of AKT, p-AKT, PI3K, and p-PI3K confirmed the activation of the PI3K-AKT pathway in response to QU treatment in AS (Figure 7D). Taken together, these data suggest that QU induces M2 polarization in AS by regulating the PI3K-AKT pathway.

## 5. Discussion

Atherosclerosis is the leading cause of cardiovascular and cerebral vessel diseases, which brings a serious burden for society and commercial development (30). To prevent and treat atherosclerosis has become an urgent demand in current medical service (31). In our study, we provided compelling evidence for the therapeutic potential of QU in atherosclerosis treatment, particularly through its ability to modulate macrophage polarization and regulate inflammatory responses. Previous studies have highlighted the critical role of macrophages in the progression of atherosclerosis, with M1 macrophages promoting inflammation and plaque instability, while M2 macrophages contribute to anti-inflammatory responses and plaque stabilization (5). Our results align with and extend these findings by demonstrating that QU induces M2 macrophage polarization, as evidenced by increased CD206 expression and upregulation of anti-inflammatory cytokines such as IL-10 and TGF-β. This is consistent with earlier reports suggesting that QU exerts immunomodulatory effects in other inflammatory diseases, such as inflammatory bowel disease and interstitial fibrosis, by targeting key signaling pathways. Notably, our study identifies the PI3K-AKT pathway as a central mechanism through which QU exerts its effects, corroborating prior research that links this pathway to M2 macrophage differentiation and anti-inflammatory responses. Furthermore, the molecular docking results, which revealed strong binding affinities of QU to key proteins such as AKT1 and JAK1, provide additional mechanistic insights into how QU may regulate macrophage function.

While previous research, such as that by Li et al. (24), has demonstrated the potential of Quercetin to modulate the PI3K/Akt pathway in acute cerebral ischemia/reperfusion injury, our study provides comprehensive evidence of this mechanism within the chronic, systemic context of atherosclerosis. Unlike the acute neuroinflammatory environment, atherosclerosis involves unique triggers like Ox-LDL and chronic lipid accumulation. Our findings specifically highlight how QU targets the PI3K-AKT axis to overcome Ox-LDL-induced cell cycle arrest and apoptosis in macrophages, offering a distinct therapeutic strategy for plaque stabilization that differs from CNS-focused interventions. Furthermore, while Tsai et al. (28) suggested that QU influences M1/M2 balance, our study integrates high-throughput sequencing with experimental verification to prove that the PI3K-AKT pathway is the crucial driver for M2 polarization in this specific vascular context. By reducing apoptosis and promoting the anti-inflammatory CD206+ phenotype, QU addresses the complex immunopathology of chronic plaque instability. These findings not only reinforce the importance of targeting macrophage polarization in atherosclerosis treatment but also position QU as a promising candidate for therapeutic development, particularly in cases where conventional lipid-lowering and anti-inflammatory therapies fall short.

A key novelty of this study lies in its integrative approach, combining big data analysis with experimental validation to uncover the mechanisms underlying QU's therapeutic effects in atherosclerosis. By leveraging network pharmacology and protein-protein interaction analysis, we systematically identified 180 potential target genes of QU in AS, which were enriched in pathways closely associated with inflammation, oxidative stress, and immune regulation. In addition, molecular docking served as a predictive hypothesis-generating tool to identify potential interaction nodes between Quercetin and atherosclerotic targets. The reliability of these computational predictions was subsequently confirmed through high-throughput RNA-sequencing and protein expression assays. Among these, the PI3K-AKT signaling pathway emerged as one of the most relevant pathways, supported by both GO and KEGG enrichment analyses. This pathway is well-documented for its role in promoting M2 macrophage polarization and resolving inflammation, making it a critical target for atherosclerosis treatment. Importantly, the findings from the big data analysis were seamlessly connected to real-world experimental results, as RNA sequencing of Ox-LDL-treated macrophages supplemented with QU confirmed significant modulation of genes within the PI3K-AKT pathway. This integrative approach not only strengthens the reliability of our findings but also underscores the power of combining computational predictions with experimental validation to uncover complex biological mechanisms. By identifying the PI3K-AKT pathway as a central mediator of QU's effects, this study provides a mechanistic framework that bridges large-scale data analysis with cellular-level insights, offering a robust foundation for future therapeutic exploration.

Despite the promising findings, this study has several limitations that warrant further investigation. First, while our in vitro experiments and bioinformatics analyses provide strong evidence for the role of QU in modulating macrophage polarization and regulating the PI3K-AKT pathway, the lack of in vivo studies limits our ability to confirm its therapeutic efficacy in a more complex physiological context. Animal models of atherosclerosis would be essential to validate the translational potential of QU and to assess its effects on plaque stability, lipid metabolism, and systemic inflammation. Second, this study did not include a parallel comparison of QU with other established treatments for atherosclerosis, such as statins or anti-inflammatory agents, which would provide a clearer understanding of QU's relative efficacy and potential advantages. Finally, while our findings highlight the PI3K-AKT pathway as a key mechanism underlying QU's effects, we did not evaluate the impact of combining QU with a PI3K-AKT inhibitor to further verify the pathway's involvement. Such experiments would provide more definitive evidence of the causal relationship between QU's therapeutic effects and PI3K-AKT signaling. While QU is promising in treating AS, the potential side effects should not be ignored. For example, it is reported that some evidence based on animal studies proved that long-term administration of QU may cause damage to pre-existing kidney diseases (32). For those with the risk of developing estrogen-dependent cancer disease, caution should be exercised when taking QU (32). However, this evidence was mainly based on animal studies, which points out the need for observational studies in human trials. Addressing these limitations in future studies will be critical to fully elucidate the therapeutic potential and mechanistic basis of QU in atherosclerosis.

### 5.1. Conclusions

**Figure 8.**
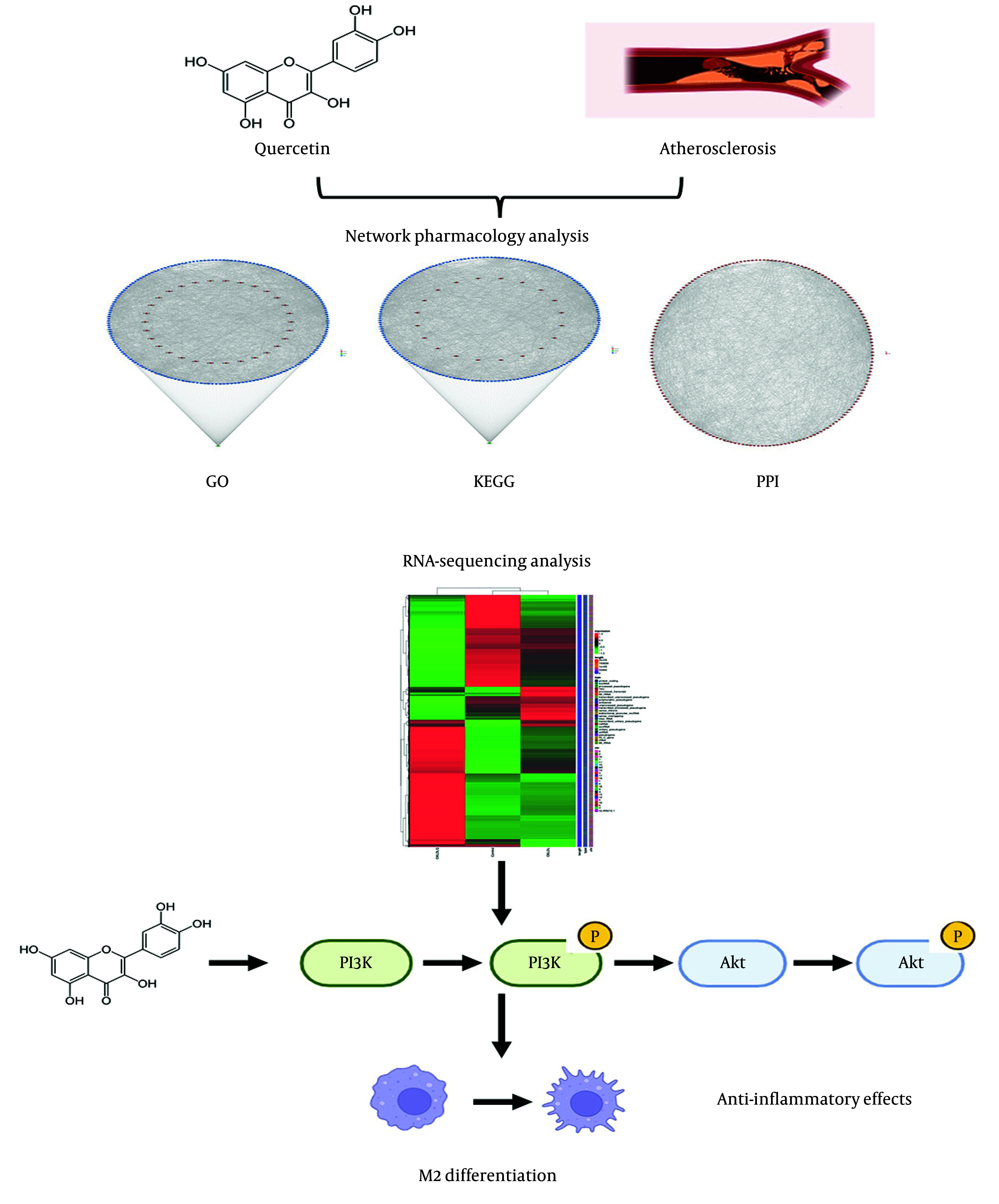
Graphical Abstract. Combinatorial analyses, including network pharmacology, protein-protein interaction studies, and RNA-sequencing, have identified the PI3K-AKT signaling pathway as a key regulatory mechanism underlying M2 macrophage differentiation, which mediates anti-inflammatory effects. These findings suggest that quercetin plays a protective role in the treatment of atherosclerosis by modulating this pathway.

In conclusion, this study highlights the potential of QU as a therapeutic agent for atherosclerosis by modulating macrophage polarization and targeting the PI3K-AKT pathway. Through bioinformatics and experimental validation, QU was shown to reduce inflammation and regulate key mechanisms in disease progression (Figure 8). While promising, further in vivo studies, comparative analyses with other treatments, and mechanistic evaluations are needed to confirm its efficacy and clarify its therapeutic role.

ijpr-25-1-168202-s001.zip

## Data Availability

The dataset presented in the study is available on request from the corresponding author during submission or after publication.
